# Public knowledge of chronic kidney disease in Portugal: a quantitative approach: Ana Rita Pedro

**DOI:** 10.1093/eurpub/ckac129.726

**Published:** 2022-10-25

**Authors:** AR Pedro, FG Avelar, B Raposo, A Boleu, S Silva, S Henriques, H Martinho

**Affiliations:** 1 Public Health Research Centre, National School of Public Health, Lisbon, Portugal; 2 Comprehensive Health Research Centre, Lisbon, Portugal; 3 Astrazeneca, Barcarena, Portugal

## Abstract

**Background:**

Chronic kidney disease (CKD) has increased progressively worldwide, however evidence on public awareness highlights gaps for a comprehensive public health strategy. This study aims to evaluate public knowledge and possible lack of awareness about CKD in Portugal.

**Methods:**

Cross-sectional study conducted through community based online survey (n = 1209). It was applied the CKD Knowledge Questionnaire and a score was calculated from 24 items categorized in 5 dimensions. Bivariate analyses was performed using one-way ANOVA and independent T-tests, at a 5% significance level, to compare the effect of independent variable and score.

**Results:**

A total of 1209 respondents with mean age 50 years (±15.1), 74.1% of female, 62.6% married or living with a partner, 55,7% at least university level. The mean (SD) public knowledge of CKD score was 14.30 (±3.4). The dimension “Functions of kidneys perform in the body” presented the lowest percentage of correct answers (48.9±19.5) while “Following are commonly used to determine health of the kidney” the highest percentage (71.3±22.4). Results of the bivariate analysis showed significant associations with gender (p < 0,001), marital status (p = 0,019), education (p < 0,001), high blood pressure (p = 0,020), personal history of kidney disease (p < 0,001), familiar history of kidney disease (p < 0,001), access to healthcare (p = 0,049), follow-up with a healthcare professional (p = 0,023), use of urgent care or hospital urgency (p = 0,020) and use of medical specialty appointments (p < 0,001).

**Conclusions:**

People living in Portugal revealed a middle knowledge level. Knowledge higher scores were observed in female, people with higher level of education, with experience imposed by health condition (e.g., risk factors or history disease), and access to health services. Future health education focused on ‘Functions of kidneys perform in the body’ might be an important contribution to increase health literacy about CKD.

**Key messages:**

• Social vulnerabilities may be associated with lower levels of public knowledge of Chronic Kidney Disease.

• The evaluation of population’s knowledge about CKD is an important instrument for public health policy-making.

